# Prevalence of presumptive clinical signs of breast cancer among women attending an early detection campaign in Lomé in 2023

**DOI:** 10.11604/pamj.2025.50.98.43237

**Published:** 2025-04-09

**Authors:** Adjovi Evenunye Sodokin, Kokoe Melinda Gbadoe, Adanvo Isaac Houngnigbe, Ben Ali, Kodjo Déla Agbewornu, Tchin Darre

**Affiliations:** 1 Medstudents Leaders Association, Lome, Togo,; 2 Euclide University Pole, Montpellier, France,; 3 Doctoral School of Health Sciences, Cotonou, Benin,; 4 Faculty of Health Sciences of Kara, Kara, Togo,; 5 Togolese Society of Obstetricians and Gynaecologists, Lome, Togo,; 6 Faculty of Health Sciences, University of Lome, Lome, Togo

**Keywords:** Prevalence, breast cancer, early detection, clinical signs, Togo

## Abstract

**Introduction:**

breast cancer is the most common cancer in women in Togo. Screening and early diagnosis campaigns are organised to limit the late use of health services. The aim of this study was to determine the prevalence of presumptive clinical signs of breast cancer and to identify associated factors in Lomé.

**Methods:**

a multicentre cross-sectional study was conducted in October 2023 in the commune of Lomé, Togo. Sampling was non-probability and voluntary. Data were collected using a digital questionnaire during the clinical examination of patients. Data were processed and analysed using R 4.3.2 software. Univariate and multivariate regression analysis were used to identify associated factors.

**Results:**

a total of 151 participants took part in the study. The largest group was aged between 18 and 24. The median age of the participants was 29 years, with an interquartile range IIQ: 23 years - 41 years. The prevalence of clinical signs of breast cancer was 35.5%. A nodule in the breast was the main symptom (24.8%), followed by breast discharge (8.3%). The number of children (ORa = 1.84; p = 0.030) was significantly associated with the presence of clinical signs of breast cancer.

**Conclusion:**

the prevalence of presumptive clinical signs of breast cancer among women during a mass early detection campaign is high. It highlights the crucial importance of early detection through mass population campaigns.

## Introduction

Breast cancer represents the most widespread neoplastic disease globally and stands as the foremost contributor to mortality associated with cancer [1]. In Togo, it constitutes the predominant malignancy, exhibiting an incidence rate of 30.7 per 100,000 females and a mortality rate of 16.7 deaths per 100,000 females attributable to cancer [2]. This mortality rate is, in part, ascribed to the advanced stage of the disease at the moment of diagnosis, with approximately 80% of individuals diagnosed presenting with either locally advanced or metastatic disease [3]. Indeed, the likelihood of survival is significantly enhanced when breast cancer is identified and treated in the early stages [4]. Despite the critical importance of early detection in improving breast cancer outcomes, numerous obstacles hinder effective screening. These include significant social, economic, and geographical barriers that restrict women's access to necessary healthcare services [5]. Analyses of cost-effectiveness have demonstrated that screening via clinical breast examination, in conjunction with comprehensive cancer care, is the most efficient approach, particularly in resource-constrained nations [6]. Current evidence suggests that, considering financial, human resources, and health infrastructure, clinical breast examination may, in the imminent future, serve as the most suitable screening strategy in sub-Saharan Africa [7]. A comprehensive referral framework and robust patient support services are essential for individuals with identified breast abnormalities to attain a timely, precise, and conclusive diagnosis.

In the absence of national initiatives, non-governmental organizations engage in awareness and early detection campaigns for breast cancer within developing nations [8]. In Togo, to mitigate the deficiency of structured screening processes, an annual breast cancer awareness and early detection initiative known as “Pink October” has been instituted since 2008 during the month of October. The primary objective of this initiative is to identify women exhibiting clinical signs and symptoms indicative of malignant breast neoplasms and facilitate their referral to specialized hospitals for accurate oncological diagnosis. In Togo, clinical presumption of breast cancer signs observed during general population early detection campaigns remains undocumented.

The aim of this research was to ascertain the prevalence of clinical indicators of breast cancer and identify correlating factors among women who participated in the 2023 edition of Pink October at the centres located in Lomé, included in our analysis.

The research questions were as follows: What is the prevalence of presumptive clinical signs of breast cancer among women attending the “Pink October” early detection campaign in Lomé, Togo, in 2023? Which clinical signs are most commonly observed during the breast examinations conducted during the campaign? What factors are associated with the presence of presumptive clinical signs of breast cancer in this population?

## Methods

**Study setting:** Togo is a state located in West Africa, with a population exceeding 8 million individuals and encompassing an area of 56,600 square kilometers. The nation is delineated into five distinct regions, with the Maritime Region serving as the location of the capital city, Lomé. Our research was conducted within the confines of Greater Lomé, which recorded a population of 2,188,376 residents in the year 2022 [9].

As reported by the National Institute of Statistics, Economic and Demographic Studies (INSEED), approximately 45.5% of the Togolese population resides beneath the threshold of poverty [10]. In Togo, a compulsory health insurance framework has been instituted for government employees and their dependents since the year 2011 [11]. Nonetheless, the rate of coverage remains exceedingly low, characterized by substantial out-of-pocket expenses, and the penetration of private health insurance is below 2% of the Togolese demographic [12]. Merely 7.6% of the Togolese population is encompassed by a social protection system. Consequently, the majority of the populace is excluded from any financial risk protection mechanisms and is compelled to incur direct health expenses through out-of-pocket payments, which are excessively burdensome [13]. This situation frequently results in individuals forgoing necessary medical treatment and resorting to self-medication practices [14].

**Study type:** this investigation was designed as a multicenter cross-sectional study with both descriptive and analytical objectives, featuring prospective data collection conducted between October 1^st^ and October 20^th^, 2023.

**Study population:** the study encompassed adult females (aged over 18 years) who presented for screening at one of the seven designated study centers, and who voluntarily consented to data collection in writing.

**Sampling:** we employed a non-probability sampling method based on voluntary participation. During the annual breast cancer early detection initiative known as “Pink October” in 2023, awareness sessions were conducted within the general populace, during which women were encouraged to participate voluntarily in examinations at the designated study centers. Those who were interested and willing to engage in the screening process were included in the study. Interested participants were then required to register either through an electronic form or via WhatsApp before attending one of the designated screening centers. Women visiting healthcare facilities were thereafter invited to partake in the study. Those who consented voluntarily and in writing were incorporated into the study cohort.

To address potential biases inherent in non-probability sampling, several strategies were implemented: i) the study was conducted across multiple centers in Lomé, which helped capture a broader demographic of women and reduced selection bias related to location; ii) participants provided informed consent before data collection, ensuring that they understood the purpose of the study and their role in it. This transparency helped build trust and encouraged participation from individuals who might have been hesitant otherwise; iii) to protect participant confidentiality and encourage honest reporting, collected data were pseudonymized. This measure aimed to mitigate social desirability bias, where participants might alter their responses based on perceived expectations; iv) the use of a structured questionnaire that included sociodemographic factors, health history, and clinical evaluations allowed for a more nuanced analysis of potential confounding variables, thus enhancing the validity of the findings.

**Data collection and variables:** data were gathered from the seven designated study centers during clinical consultations, which encompassed both an interview and a physical examination of the patients' breasts. The study utilized a digitalized questionnaire administered via KoboToolBox. Prior to full deployment, the questionnaire underwent pilot testing with a small group of participants from the target population. This step was crucial for assessing the clarity, relevance, and cultural appropriateness of the questions. Feedback from the pilot test was used to refine the questionnaire to ensure that it effectively captured the necessary data. Interviewers underwent comprehensive training sessions before data collection commenced. The interviewers were primarily medical practitioners. Their professional training equipped them with the necessary skills to conduct clinical examinations and interviews effectively. The interviewers had prior experience working in community health settings or had participated in similar screening campaigns. This experience contributed to their understanding of the local context and cultural sensitivities surrounding breast cancer screening.

The scope of data collection encompassed sociodemographic attributes, healthcare accessibility, personal and familial antecedents of breast cancer, comorbid conditions, modifiable risk factors (including physical activity, tobacco use, and alcohol intake), as well as knowledge, attitudes, and practices pertinent to breast cancer, alongside data derived from the physical examinations conducted by the medical professionals. Physical activity levels were evaluated through the Marshall questionnaire [15]. The dependent variable was defined as the presence of a presumptive clinical indicator of breast cancer, categorized as “yes” or “no”. A presumptive clinical indicator of breast cancer was deemed present in a participant when a medical practitioner objectively discerned it during a clinical assessment.

**Statistical methods:** data were processed and analyzed employing R software version 4.3.2. To estimate the prevalence of presumptive clinical signs of breast cancer, we divided the number of participants exhibiting at least one presumptive clinical sign indicative of breast cancer by the total number of participants examined. Proportions were compared utilizing Pearson's chi-squared test or Fisher's exact test. Both univariate and multivariate regression analyses were conducted to elucidate factors correlated with the presence of presumptive clinical indicators of breast cancer. Participants who did not complete the clinical examination were excluded from certain analyses to ensure the integrity and validity of the study's findings.

**Ethical considerations:** the study secured authorization from the governing bodies of the centers engaged in data collection. Informed and written consent was obtained from the participants prior to the initiation of data collection. The study adheres to the principles of medical confidentiality concerning the women who participated in the survey. The data collected was pseudonymized to safeguard the anonymity and confidentiality of the information, which is exclusively utilized for scientific research endeavors.

## Results

### Description of the sample

**Sociodemographic characteristics:** a total of 151 female participants, with ages ranging from 18 to 69 years, were incorporated into the study sample. The demographic cohort aged 18 to 24 years represented the predominant segment, accounting for 35.8% (Table 1). The median age of the participants was established at 29 years, with an interquartile range (IQR) of (23 years - 41 years).

**Table 1:** distribution of respondents based on socio-demographic characteristics, Lomé, 2023

	Frequency (N = 151)	Percentage
**Age**		
18 - 24	54	35.8
25 - 34	43	28.5
35 - 44	20	13.2
45 - 54	17	11.3
≥ 55	17	11.3
**Marital status**		
Single	81	53.6
Married	70	46.4
**Child status**		
No	79	52.3
Yes	72	47.7
**Number of children**		
< 4	55	76.4
≥ 4	17	23.6
**Educational level**		
None	18	11.9
Secondary	80	53.0
Primary	16	10.6
Higher	37	24.5
**Income-generating activity**		
No	54	35.8
Yes	97	64.2
**Monthly income level**		
≥ Minimum wage	35	36.1
< Minimum wage	62	63.9
**Healthcare professional**		
No	144	95.4
Yes	7	4.6
**Health insurance coverage**		
No	124	82.1
Yes	27	17.9

A significant portion of the population identified as single (53.6%). Approximately half of the population (47.7%) reported having children, exhibiting a relatively balanced distribution between those with two or fewer children and those with more than two. A substantial majority of the population had achieved a secondary level of education (53%). A considerable portion of the population (64.2%) was engaged in activities that generated income. The analysis of monthly income levels indicated a notable predominance (63.9%) of earnings below the locally established minimum wage (MW) of 52,500 CFA francs in 2023. A minor fraction of the population (4.6%) was employed within the healthcare sector. The majority of the population (82.1%) lacked health insurance or any form of coverage.

**History, comorbidities, and modifiable risk factors:** Table 2 delineates the profile of the study population predicated on historical data and comorbid conditions. In terms of smoking behavior, a vast majority (97.4%) of participants indicated that they were non-smokers. Concerning alcohol consumption, 59.6% of individuals acknowledged the intake of alcoholic beverages. Regarding physical activity levels, an overwhelming majority (86.1%) were classified as insufficiently active. One in four participants displayed characteristics indicative of obesity. More than 80% of participants reported the absence of chronic diseases. Lastly, with respect to family history of breast cancer, a significant majority (85.4%) reported no such history, while 14.6% indicated a familial occurrence.

**Table 2:** distribution of respondents based on history and comorbidities, Lomé, 2023

	Frequency (N = 151)	Percentage
**Smoking**		
No	147	97.4
Yes	4	2.6
**Alcohol consumption**		
Yes	90	59.6
No	61	40.4
**Physical activity**		
Insufficiently active	130	86.1
Sufficiently active	21	13.9
**BMI**		
Normal	74	49.0
Overweight	40	26.5
Obesity	37	24.5
**Chronic diseases**		
No	125	82.8
Yes	26	17.2
**Family history of breast cancer**		
No	129	85.4
Yes	22	14.6

**Prevalence of presumptive clinical signs of breast cancer:** the participants of the survey underwent clinical breast examinations conducted by medical professionals at designated centers. Data from these clinical evaluations were systematically recorded for 121 participants. The clinical signs assessed included the presence of nodules in the breast, discharge from the breast, deformation or swelling of the breast/nipple, alterations in the skin of the breast/nipple, presence of lymph nodes in the axillary region, atypical breast pain, and soreness of the breast/nipple. Among the 121 participants evaluated, 43 exhibited at least one presumptive clinical sign indicative of breast cancer, culminating in a prevalence rate of 35.5%.

Among the clinical manifestations identified in this cohort of 43 women, the presence of nodules emerged as the most prevalent symptom (69.8%; n=30), followed by breast discharge (23.3%; n=10) and atypical breast pain (21.0%; n=9). The incidence rates of these three predominant symptoms within the study population were recorded at 24.8%, 8.3%, and 7.4%, respectively. The remaining clinical indicators exhibited minimal (<5%) or nonexistent prevalence, most notably breast or nipple soreness.

Table 3 delineates the distribution of participants categorized by the clinical indicators of breast cancer and the affected breast. Among participants exhibiting nodules in the breast (24.8%), a predominant proportion was found to involve the right breast (50.0%). In the case of those experiencing breast discharge (23.3%), the distribution was observed across the right breast (20.0%), left breast (30.0%), and bilateral involvement (50.0%). Concerning breast/nipple deformation or swelling (11.6%), a significant majority of cases pertained to the right breast (60.0%). Alterations in the skin of the breast/nipple (4.7%) were exclusively documented in the right breast (100.0%). For the occurrence of lymphadenopathy in the axillary region (4.7%), all instances were localized to the right axillary area (100.0%). Finally, among individuals reporting atypical breast pain (20.9%), the right breast predominantly exhibited symptoms (44.4%).

**Table 3:** distribution of respondents based on the prevalence of presumptive clinical signs of breast cancer and the affected breast, Lomé, 2023

	Frequency (N = 121)	Percentage
Breast nodules	30	24.8
Right breast	15	50.0
Left breast	10	33.3
Both breasts	5	16.7
Breast discharge	10	8.3
Right breast	2	20.0
Left breast	3	30.0
Both breasts	5	50.0
Breast deformation/swelling	5	4.1
Right breast	3	60.0
Left breast	2	40.0
Changes in breast/nipple skin	2	1.7
Right breast	2	100.0
Presence of armpit lymph nodes	2	1.7
Right armpit	2	100.0
Unusual breast pain	9	7.4
Right breast	4	44.4
Left breast	2	22.2
Both breasts	3	33.3

Although a significant majority of participants (53.0%; n = 80) indicated engagement in breast self-examination (BSE), only a mere 9.3% reported performing this practice consistently. In terms of mammography, 78.1% (n = 118) of the population disclosed that they had never undergone this diagnostic procedure.

Of the 43 participants exhibiting clinical signs, merely 24 (55.8%) had previously identified this anomaly through breast self-examination (BSE). It is noteworthy that a substantial majority (91.6%; n = 21) of these anomalies had been recognized at least 6 months prior during BSE. Nevertheless, 45.8% (n = 11) had never sought medical consultation for this concern, and only a minority (33.3%; n = 8) managed to consult a physician within two months following the identification of anomalies. Among the 24 individuals who recognized an anomaly via breast self-examination, 14 (58.3%) sought medical consultation.

Of the 43 participants, most participants (37.2%; n = 16) were aged between 18 and 24 years. The median age of the participants was established at 30 years, with an interquartile range (IQR) of 24 years to 48 years. A slight majority (51.2%; n = 22) had no children. The majority (53.5%; n = 23) had attained secondary education. Most participants (41.9%; n =18) earned less than the minimum wage.

**Factors associated with the presence of presumptive clinical signs of breast cancer:** Table 4 delineates the distribution of odds ratios (OR) pertaining to factors correlated with the manifestation of presumptive clinical signs indicative of breast cancer, analyzed through both univariate and multivariate methodologies. The presence or absence of children demonstrated no statistically significant correlation with presumptive clinical signs of breast cancer in the univariate analysis (p = 0.7). Conversely, the presence of four or more children attained statistical significance in the multivariate analysis (OR = 5.64; p = 0.029). Age did not exhibit statistical significance (p = 0.12). Furthermore, no notable association was identified between the level of education and the presumptive clinical signs of breast cancer (p = 0.7).

**Table 4:** distribution of odds ratios of factors for the presence of presumptive clinical signs of breast cancer in univariate and multivariate analysis

			Univariate analysis			Multivariate analysis		
	N	n (%)	ORc	95% CI	p-value	ORa	95% CI	p-value
**Age**					**0.12**			**0.3**
18 - 24	40	16 (40.0%)	-	-		1	-	
25 - 34	34	7 (20.6%)	0.39	0.13 - 1.07	0.076	0.18	0.01 - 5.18	0.2
35 - 44	19	8 (42.1%)	1.09	0.35 - 3.30	0.9	0.88	0.06 - 24.3	>0.9
45 - 54	14	4 (28,6%)	0.6	0.14 - 2.14	0.4	0.34	0.02 - 10.5	0.5
≥ 55	14	8 (57.1%)	2	0.59 - 7.15	0.3	1.12	0.06 - 35.0	>0.9
**Child status**					**0.7**			
No	59	22 (37.3%)	-	-				
Yes	62	21 (33.9%)	0.86	0.41 - 1.82	0.7			
**Number of children**					**0.009**			**0.022**
< 4	50	13 (26.0%)						
≥ 4	12	8 (66.7%)	5.69	1.53 - 24.4	0.012	5.64	1.27 - 29.9	0.029
**Educational level**					**0.7**			
None	17	8 (47.1%)	-	-				
Secondary	65	23 (35.4%)	0.62	0.21 - 1.85	0.4			
Primary	11	4 (36.4%)	0.64	0.13 - 2.99	0.6			
Higher	28	8 (28.6%)	0.45	0.12 - 1.58	0.2			
**Monthly income level**					**0.4**			
≥ Minimum wage	31	9 (29.0%)	-	-				
< Minimum wage	48	18 (37.5%)	1.47	0.56 - 3.99	0.4			
**Smoking**					**>0.9**			
No	118	42 (35.6%)	-	-				
Yes	3	1 (33.3%)	0.9	0.04 - 9.71	>0.9			
**Alcohol consumption**					**0.4**			
Yes	74	24 (32.4%)	-	-				
No	47	19 (40.4%)	1.41	0.66 - 3.03	0.4			
**Regular physical activity**					**0.5**			
Insufficiently active	102	35 (40.8%)	-	-				
Sufficiently active	19	8 (31.9%)	0.68	0.32 - 1.45	0.3			
**BMI**					**0.4**			
Normal	58	24 (41.4%)	-	-				
Overweight	34	10 (29.4%)	0.59	0.23 - 1.43	0.3			
Obesity	29	9 (31.0%)	0.64	0.24 - 1.61	0.4			
**Chronic diseases**					**0.7**			
No	99	36 (36.4%)	-	-				
Yes	22	7 (31.8%)	0.82	0.29 - 2.13	0.7			
**Family history of breast cancer**					**0.2**			**0.6**
No	103	39 (37.9%)	-	-		1	-	
Yes	18	4 (22.2%)	0.47	0.13 - 1.42	0.2	1.62	0.30 - 9.00	0.6

Factors such as smoking, alcohol consumption, regular physical activity, body mass index (BMI), and chronic diseases revealed no significant associations with presumptive clinical signs of breast cancer (p > 0.3). In a similar vein, a familial history of breast cancer was not found to be associated with the presence of presumptive clinical signs of breast cancer.

## Discussion

**Prevalence of clinical signs in the study population:** this investigation, to the best of our understanding, represents the inaugural effort undertaken in Togo to evaluate the prevalence of presumptive clinical indicators of breast cancer among women during a widespread early detection initiative. A prospective, more comprehensive investigation encompassing both metropolitan and rural locales, as well as a diverse age demographic, would serve to further enhance these findings. Indeed, the cohort involved in our study exhibits a selection bias, as it is primarily composed of younger individuals, which consequently impacts the outcomes. A retrospective analysis of all histologically confirmed breast cancer cases among women over a two-decade span in Togo indicated that the median age of diagnosis was 46.7 years [16]. Therefore, it is reasonable to assert that the prevalence of presumptive clinical signs observed in our sample may be an underrepresentation when contrasted with a more age-inclusive population. While our research did not identify a statistically significant correlation between age and the manifestation of clinical signs, a larger-scale investigation could yield a more substantial statistical power to examine this association.

In our investigation, the occurrence of nodules and breast discharge emerged as the predominant presumptive clinical signs, corroborating the findings of the study conducted by Darré *et al*. [3]. Their examination, conducted at the Sylvanus Olympio Teaching Hospital, revealed that 75.8% of consultations were prompted by the presence of a nodule, with breast discharge following at a rate of 30.6%.

While our investigation documented a prevalence of presumptive clinical indicators of breast cancer at 35.5%, it is imperative to acknowledge that not all such indicators are necessarily indicative of malignancy. Indeed, the research conducted by Amadou *et al*. which focused on mammographic outcomes during screening campaigns in Lomé over a five-year timeframe, demonstrated that lesions classified as suspicious for malignancy (BI-RADS IV) and highly suggestive of malignancy (BI-RADS V) constituted 3.5% and 1.9% of cases, respectively, leading to a cumulative prevalence of 5.4% [17]. A subsequent study that integrates the prevalence of presumptive clinical signs within the general populace alongside the prevalence of malignant cases would furnish additional insights to augment our findings.

**Association between the number of children and presumptive clinical signs of breast cancer:** our investigation revealed a significant correlation between the quantity of offspring and the manifestation of indicative signs of breast cancer. Specifically, women who had borne more than four children exhibited a fivefold increase in the probability of displaying presumptive clinical signs of breast cancer when juxtaposed with their counterparts who had fewer than four children. Nonetheless, due to the cross-sectional nature of our research as opposed to a prospective design, there exists an ambiguity in ascertaining the exact timing of the emergence of these anomalies. It is plausible that certain women with more than four children may have experienced the development of these anomalies several years prior to their detection, particularly in light of the notably low incidence (9.27%) of regular breast self-examination among the study participants.

**Detection of breast anomalies and medical consultation delay:** the findings from our research underscore the alarmingly low frequency of the regular practice of breast self-examination (BSE) and mammographic screening within the population under study, which consequently hampers the early identification of anomalies. In the context of sub-Saharan Africa, it has been evidenced that possessing health insurance coverage and a heightened socioeconomic status correlate positively with breast cancer screening engagement [18]. Given the markedly limited health coverage prevalent among the Togolese populace, which is predominantly characterized by poverty, it is of paramount importance to implement free screening initiatives aimed at facilitating the early detection of the disease. Furthermore, it is essential to augment awareness campaigns directed at women to encourage the consistent practice of BSE.

Within our study cohort, we also observed that the pursuit of medical consultation upon the detection of anomalies was not a systematic practice among women, and when such consultations did occur, they were frequently delayed. It remains vital to enhance awareness among Togolese women regarding the indicative signs and symptoms of breast cancer and the necessity for prompt medical consultation upon detection. Indeed, Togolese women diagnosed with breast cancer often present at advanced stages of the disease, with contributing factors to this delay including apprehension regarding diagnosis, the absence of breast self-examination practices, and the inclination to resort to traditional remedies and self-medication as initial responses [3].

Moreover, a study conducted by Djahini *et al*. indicates that the likelihood of seeking consultation from a general practitioner in Togo is statistically elevated in the Maritime region. Similarly, residing in the Grand Lomé region correlates with a higher probability of consulting a specialist physician compared to those living in the Maritime Region [12]. Thus, the delays in consultation noted within our study population may be underestimated in comparison to populations residing in rural locales.

Although our study population is exclusively situated in Lomé and its surrounding areas, where healthcare facilities are more accessible than in rural settings, our findings reveal that over 80% of respondents lack health coverage, and more than 60% possess incomes below the local minimum wage. These results illuminate potential justifications for the abandonment of healthcare services, accentuating the pressing need for the establishment of universal health coverage in Togo. This necessity is further underscored by the fact that the direct costs associated with breast cancer treatment significantly exceed the average household income in Togo [19].

**Modifiable risk factors:** in addition to the consumption of alcoholic beverages, our research identifies the deficiency of physical activity as a predominant modifiable risk factor affecting the majority of participants. Indeed, over 80% of the respondents exhibited insufficient levels of physical activity, thus underscoring the necessity for enhanced public education regarding modifiable risk factors. A preceding study conducted by Ketevi *et al*. among Togolese women indicated that, while a majority acknowledge the existence of breast cancer, their awareness of its signs and associated risk factors remains markedly constrained [20]. Consequently, addressing modifiable risk factors is essential for enhancing the preventive measures against this disease.

**Study perspectives:** in the context of our investigation, we did not assess the diagnostic approaches employed by practitioners when confronted with identified cases during screening, nor did we evaluate the diagnoses established subsequent to histopathological examination. An analysis of diagnostic methodologies and patient follow-up could represent a valuable avenue for further research. Moreover, in light of the inadequate health coverage available to numerous women, some individuals may be unable to afford the diagnostic tests that are prescribed. A study that investigates the rate at which healthcare is abandoned for financial reasons would significantly contribute to our understanding of the impact that the absence of health coverage has on these women's access to medical care.

**Limitations:** the study employed a non-probability sampling method based on voluntary participation. While this approach can introduce selection bias, efforts were made to mitigate its impact. Prior to the screening, awareness sessions were conducted to educate women about breast cancer and the importance of early detection. This outreach aimed to encourage participation from a diverse group of women rather than just those already engaged with healthcare services. Interested participants were required to register in advance through an electronic form or WhatsApp. This process helped ensure that a wider demographic was reached, including those who might not typically seek screening. The study was conducted across multiple centers in Lomé, allowing for the inclusion of women from various socioeconomic backgrounds and geographical areas. This diversity aimed to reflect the broader population and minimize biases associated with specific locations. Univariate and multivariate regression analyses were employed to identify factors associated with the presence of clinical signs while controlling for confounding variables. This statistical approach helps clarify relationships between variables and reduces biases that could arise from uncontrolled factors.

Another study limitation is that our research was limited to the city of Lomé and its surrounding areas, which may restrict the generalizability of the findings to rural regions with less accessible healthcare services. Furthermore, we did not evaluate the proportion of malignant cases. It is imperative to acknowledge that, despite identifying a 35.5% prevalence of clinical signs indicative of breast cancer, not all of these signs denote malignancy.

## Conclusion

This study, conducted during the “Pink October” campaign in Lomé, Togo, in 2023, revealed a high prevalence (35.5%) of presumptive clinical signs of breast cancer among participants, underscoring the critical importance of early detection campaigns. Breast nodules were the most common symptom (24.8%), followed by breast discharge (8.3%). Multivariate analysis identified a significant association between having four or more children and the presence of clinical signs (ORa = 5.64; p = 0.029). While 55.8% of women with clinical signs had detected them through self-examination, only 33.3% consulted a physician within two months, revealing significant barriers to care access. These findings highlight the need for improved breast self-examination education, expanded access to healthcare services, and strengthened breast cancer screening programs in Togo. Future research should focus on evaluating the long-term impact of early detection campaigns and exploring cultural and socioeconomic barriers to seeking medical care. This study emphasizes the urgent need for comprehensive, accessible, and culturally appropriate breast cancer screening strategies in resource-limited settings to reduce the burden of late-stage diagnoses and improve outcomes.

### What is known about this topic

- Togolese women with breast cancer often have a delayed presentation, leading to advanced-stage diagnoses;
- Breast nodules and breast discharge are the common reasons for consultations.

### What this study adds

- This study highlights a significant prevalence of presumptive clinical signs of breast cancer among women participating in an early detection campaign in Lomé, with a reported prevalence of 35.5%;
- The research identifies specific factors associated with the presence of clinical signs of breast cancer, such as the number of children, which was found to have a significant association.
